# Supplementary figures and images for: The COVID-19 Pandemic and Mental Health Concerns on Twitter in the United States
Source: Health Data Sci. 2022 Feb 17;2022:9758408. doi: 10.34133/2022/9758408 (PMC9629680; doi:10.34133/2022/9758408)

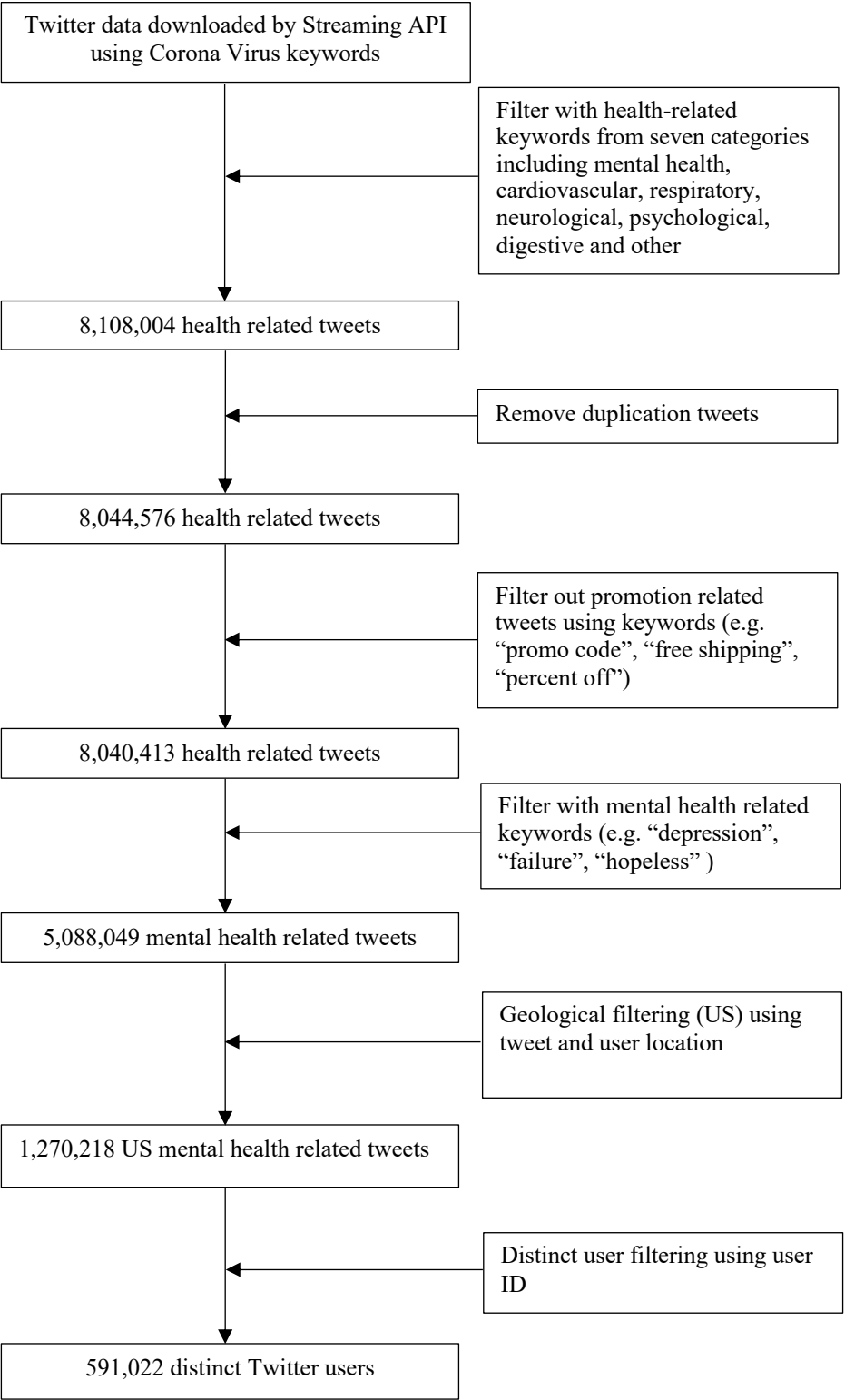

Supplement: Supplementary 1 — Appendix Figure 1: flow chart of data preprocessing. [file 9758408.f1.pdf]

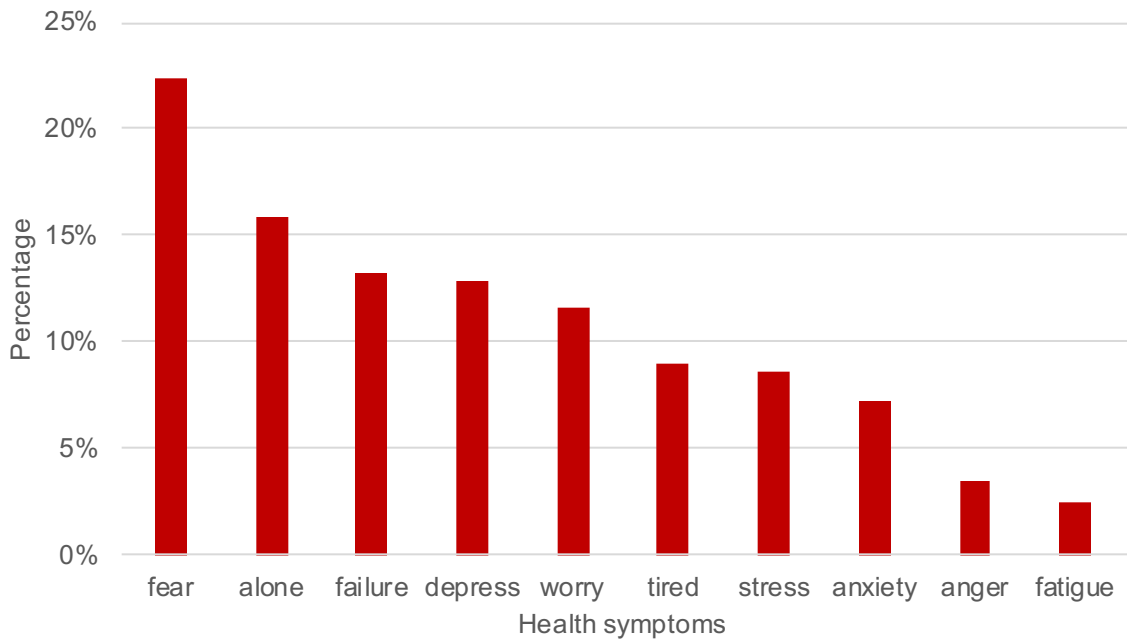

Supplement: Supplementary 2 — Appendix Figure 2: mental health-related keywords that were mentioned the most in COVID-19-related tweets in the US. [file 9758408.f2.pdf]

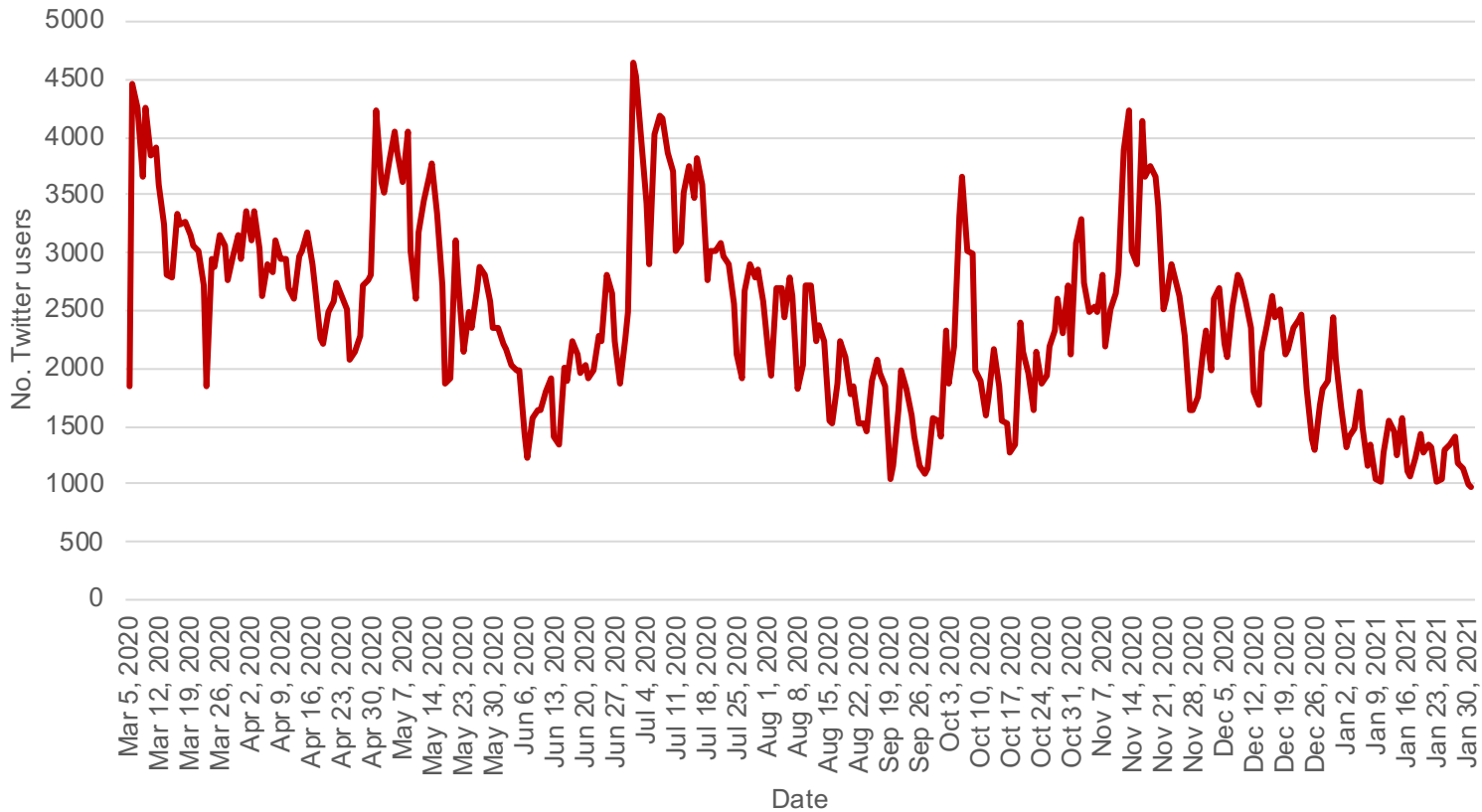

Supplement: Supplementary 3 — Appendix Figure 3: number of Twitter users who had mental health concerns over time in the US. [file 9758408.f3.pdf]

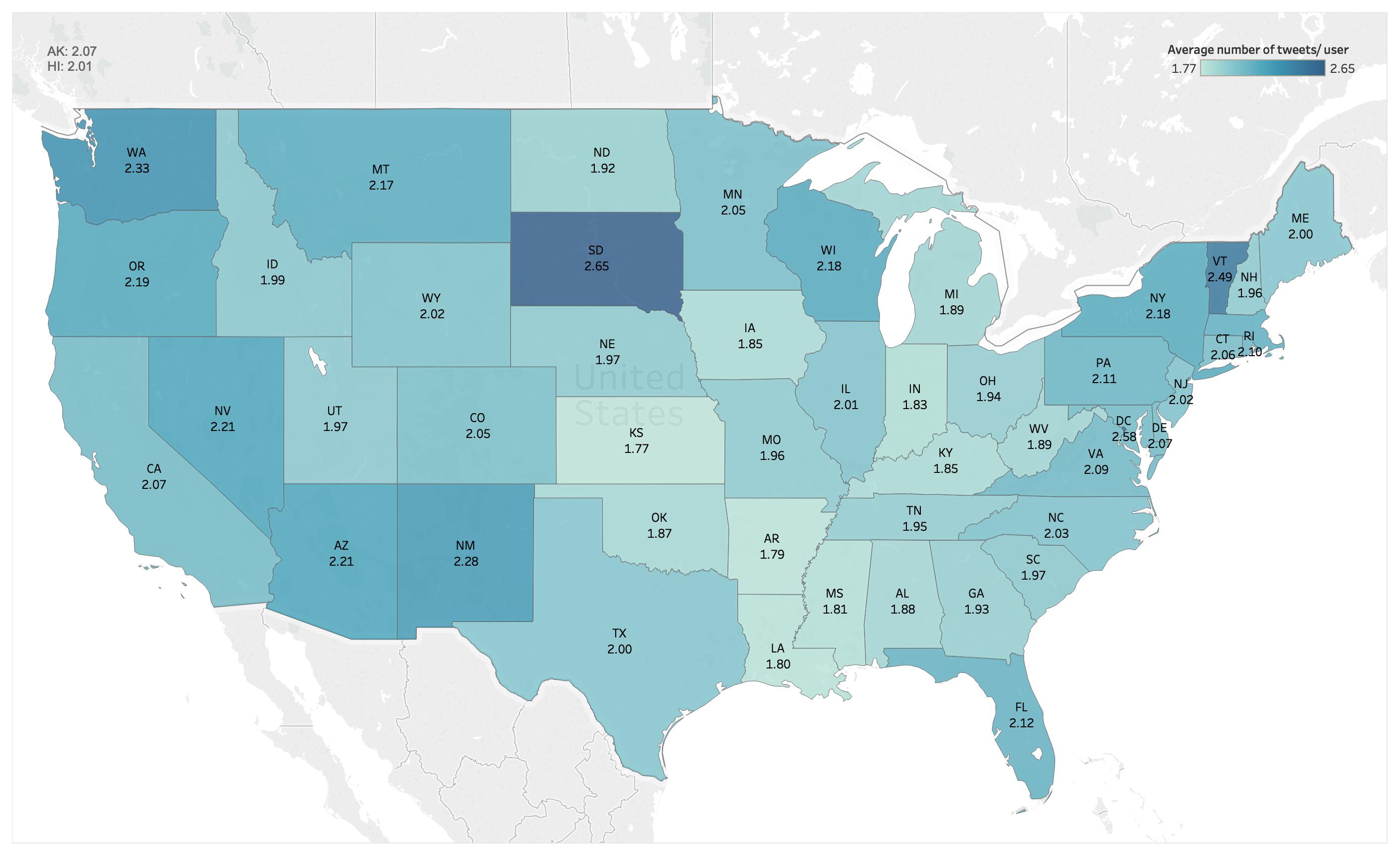

Supplement: Supplementary 4 — Appendix Figure 4: average number of mental health-related tweets per user in different US states. [file 9758408.f4.jpg]

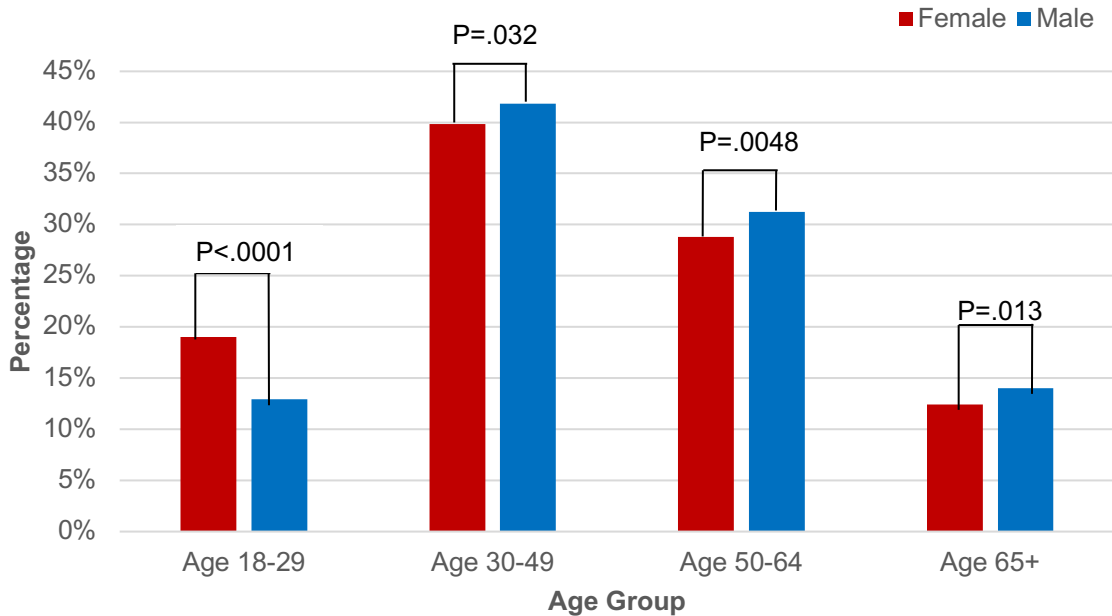

Supplement: Supplementary 5 — Appendix Figure 5: age composition in different gender groups for Twitter users who had mental health concerns in the US. [file 9758408.f5.pdf]

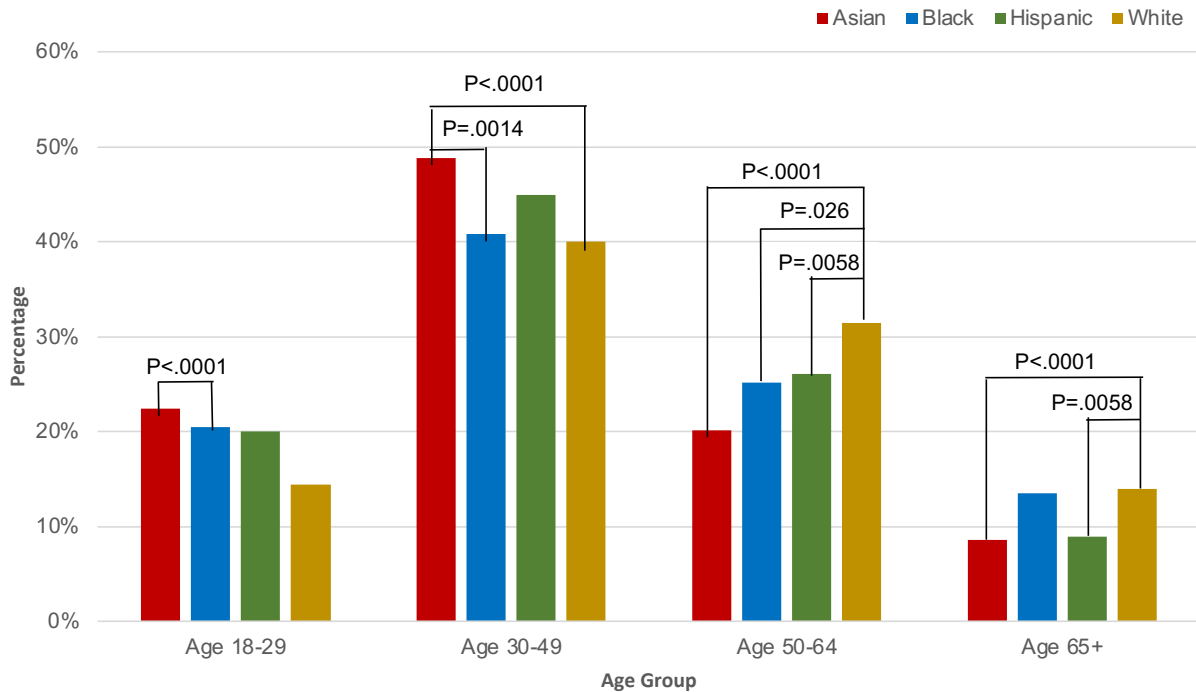

Supplement: Supplementary 6 — Appendix Figure 6: age composition in different race/ethnicity groups for Twitter users who had mental health concerns on Twitter in the US. [file 9758408.f6.pdf]
